# Neutralisation of SARS‐CoV‐2 by anatomical embalming solutions

**DOI:** 10.1111/joa.13549

**Published:** 2021-10-11

**Authors:** Fabio Quondamatteo, Dora E. Corzo‐Leon, Cecilia Brassett, Ian Colquhoun, David C. Davies, Peter Dockery, Sue Grenham, Simon Guild, Amanda Hunter, James Jones, Thomas C. Lee, Chris Tracey, Tracey Wilkinson, Carol A. Munro, Thomas H. Gillingwater, Simon H. Parson

**Affiliations:** ^1^ Department of Anatomy and Regenerative Medicine Royal College of Surgeons in Ireland Dublin Ireland; ^2^ School of Medicine Medical Sciences and Nutrition University of Aberdeen Aberdeen UK; ^3^ Human Anatomy Centre Department of Physiology, Development and Neuroscience University of Cambridge Cambridge UK; ^4^ Royal College of Physicians and Surgeons of Glasgow Glasgow UK; ^5^ Human Anatomy Unit Department of Surgery and Cancer Imperial College London London UK; ^6^ Anatomy School of Medicine NUI Galway Galway Ireland; ^7^ Department of Anatomy and Neuroscience University College Cork Cork Ireland; ^8^ School of Life Sciences University of Glasgow Glasgow UK; ^9^ Anatomy Centre ARU Medical School Anglia Ruskin University Chelmsford UK; ^10^ Biomedical Section School of Medicine University College Dublin Dublin Ireland; ^11^ Centre for Anatomy and Human Identification School of Science and Engineering University of Dundee Dundee UK; ^12^ Anatomy Edinburgh Medical School: Biomedical Sciences College of Medicine and Veterinary Medicine University of Edinburgh Edinburgh UK

**Keywords:** body donation, cadaver, fixation, preservation

## Abstract

Teaching and learning anatomy by using human cadaveric specimens has been a foundation of medical and biomedical teaching for hundreds of years. Therefore, the majority of institutions that teach topographical anatomy rely on body donation programmes to provide specimens for both undergraduate and postgraduate teaching of gross anatomy. The COVID‐19 pandemic has posed an unprecedented challenge to anatomy teaching because of the suspension of donor acceptance at most institutions. This was largely due to concerns about the potential transmissibility of the SARS‐CoV‐2 virus and the absence of data about the ability of embalming solutions to neutralise the virus. Twenty embalming solutions commonly used in institutions in the United Kingdom and Ireland were tested for their ability to neutralise SARS‐CoV‐2, using an established cytotoxicity assay. All embalming solutions tested neutralised SARS‐CoV‐2, with the majority of solutions being effective at high‐working dilutions. These results suggest that successful embalming with the tested solutions can neutralise the SARS‐CoV‐2 virus, thereby facilitating the safe resumption of body donation programmes and cadaveric anatomy teaching.

## Graphical abstract

Twenty different, commonly used anatomical embalming solutions neutralise SARS‐CoV‐2, at a range of dilutions.

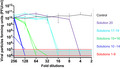

## 1 INTRODUCTION

The use of human cadaveric specimens for anatomy teaching remains a cornerstone of medical and scientific education (Ghosh, 2017). There are currently 47 medical schools in the United Kingdom and Ireland, and at least 2600 worldwide (Duvivier et al., 2014), the vast majority of which rely upon body donation and embalming preservation techniques (Habicht & Kiessling, 2018). The ongoing COVID‐19 pandemic resulted in immediate, widespread cessation of the acceptance of donor bodies by medical schools and universities, largely because of the perceived risks to staff and students (Brassett et al., 2020). Moreover, the challenge of community‐based testing as well as the poor specificity and sensitivity of some tests meant that there could not be any guarantee that accepted donors were virus‐free (Morgan et al., 2021). Critically, there have been no data published about the efficacy of anatomical embalming solutions to neutralise SARS‐CoV‐2. Such data are urgently required in order to inform decisions about the safe resumption of body donation programmes and cadaver‐based anatomy education. Therefore, in the current study, the ability of 20 different, bespoke and commercial anatomical embalming solutions used in the United Kingdom and Ireland, to neutralise SARS‐CoV‐2, was tested. All 20 solutions tested were effective, several of them at high dilutions, resulting in effective neutralisation of anti‐SARS‐CoV‐2 at concentrations well below those used to embalm a body.

## 2 MATERIAL AND METHODS

### 2.1 Cell cultures

Vero E6 (ATCC^®^ CRL‐1586™) cells were obtained from the MRC‐University of Glasgow Centre for Virus Research. Cells were maintained at 37°C and 5% CO_2_ in Dulbecco Modified Eagle Medium (DMEM) supplemented with 10% foetal calf serum (FCS) and 1% penicillin/streptomycin.

### 2.2 Viral cultures

SARS‐CoV‐2 Strain England 2 was obtained from Public Health England and propagated in Vero E6 cells for 72 h at 37°C and 5% CO_2_ in DMEM supplemented with 2% FCS and 1% penicillin/streptomycin. After 72 h, the resulting supernatants were recovered and stored at −70°C. Viruses from second and third cell passages were used in all experiments.

#### 2.2.1 Determination of cytotoxicity levels for each embalming solution

Vero E6 cells of 3 × 10^4^ in 10% FCS supplemented DMEM were added to each well of a 96‐well plate and incubated for 24–36 h at 37°C and 5% CO_2_. On the day of the experiment, two‐fold dilutions (1:2–1:8) were carried out for each embalming solution using DMEM supplemented with 2% FCS. Each two‐fold dilution was further diluted 10‐fold (10^1^–10^7^) in 2% FCS supplemented DMEM to obtain a final volume of 50 µl/dilution. Samples of the diluted embalming solution were added to individual wells of a 96‐well plate containing Vero E6 cells (described above) and incubated for 1 h at 37°C and 5% CO_2_. After 1 h, 100 µl of DMEM supplemented with 2% FCS was added to each well and the plate was incubated for 72 h at 37°C and 5% CO_2_. After 72 h, cells were fixed for 1 h with 10% neutral‐buffered formalin and then stained with 1% crystal violet (CV) in 20% ethanol. Safe dilutions were those at which the embalming solution was not toxic to Vero E6 cells.

### 2.3 Viral microtitration

Vero E6 cells were cultured in 96‐well plates as described for the cytotoxicity assay above. Each embalming solution was diluted two‐fold (1:2–1:128) in 2% FCS supplemented DMEM in a final volume of 50 µl. The viral inoculum was prepared in 2% FCS supplemented DMEM at a range of 4.5 × 10^8^–4.5 × 10^10^ PFU/ml. A 50 µl aliquot of viral inoculum (~2.3 × 10^9^ PFU) was added to 50 µl diluted embalming solution and incubated for 1 h at 37°C and 5% CO_2_. After this incubation period, each diluted embalming solution w/virus was further diluted 10‐fold (10^1^–10^10^) in 2% FCS supplemented DMEM. Depending on the cytotoxicity, 50 µl samples of each dilution from 10^4^ or 10^5^ to 10^9^ or 10^10^ were transferred into wells of 96‐well plates containing the previously prepared 3 × 10^4^ Vero E6 cells. Then, Vero cells plus embalming solution/virus were incubated for 1 h at 37°C and 5% CO_2_ (adsorption phase). After the adsorption phase, 100 µl of 1.2% Avicel^®^ PH‐101 solution in 2% FCS DMEM were added to each well before the cells were incubated again for 72 h at 37°C and 5% CO_2_. After 72 h, the cells were fixed in 150 µl of 10% neutral buffered formalin for 3 h before being stained with 1% CV in 20% ethanol solution. After staining, plaques were quantified, and viral titres were determined as previously published (Baer & Kehn‐Hall, 2014). Each embalming solution and its two‐fold dilutions were tested in three independent experiments.

## 3 RESULTS AND DISCUSSION

In order to rule out a potential direct cytotoxic effect of the embalming solutions tested on the Vero E6 cell system, dilutions of the embalming solutions that were non‐toxic to Vero E6 cells were determined. The non‐toxic dilutions for each embalming fluid tested (safe dilution) are listed in Table 1. This eliminated any potential cytotoxic effects of the embalming solutions on the Vero E6 cells and ensured that data obtained would be based solely upon the ability of an embalming solution to neutralise the virus.

**TABLE 1:** Safe dilution, chemicals, pH and range of SARS‐CoV‐2 neutralisation of each embalming solution tested

Embalming solution	Safe dilution	pH	Formaldehyde (approx.)	Pure phenol	Ethanol	Industrial denatured alcohol	Methanol	Glycerol	PEG	3‐log10 reduction inhibitory dilutions (concentrations)
1	10⁶	5	2.8%	6.1%	58.4%	N/A	3.1%	N/A	N/A	1:2–1:256 (50%–0.39%)
2	10⁶	5	4%	6.1%	58.4%	N/A	3.1%%	N/A	N/A	1:2–1:256 (50%–0.39%)
3	10⁷	10	9%	*	*	*	*	*	*	1:2–1:256 (50%–0.39%)
4	10⁵	8	N/A	*	*	*	*	*	*	1:2–1:256 (50%–0.39%)
5	10⁵	5	4.2%	N/A	38%	N/A	1.5%	N/A	N/A	1:2–1:256 (50%–0.39%)
6	10⁶	5	2.5%	3.5%	N/A	N/A	77.43%	11.5%	N/A	1:2–1:256 (50%–0.39%)
7	10⁴	11	6.5%	*	*	*	*	*	*	1:2–1:256 (50%–0.39%)
8	10⁴	9	9%	*	*	*	*	*	*	1:2–1:256 (50%–0.39%)
9	10⁵	5	3.75%	8.57%	N/A	N/A	57.37%	21.43%	N/A	1:2–1:256 (50%–0.39%)
10	10⁵	5	3.18%	3.63%	N/A	72.92%	N/A	N/A	9.09%	1:2–1:128 (50%–0.78%)
11	10⁵	6	1.86%	*	*	*	*	*	*	1:2–1:128 (50%–0.78%)
12	10⁵	5	2.19%	2.5%	N/A	N/A	78.26%	12.5%	N/A	1:2–1:128 (50%–0.78%)
13	10⁵	5	2.625%	4%	N/A	62.65%	N/A	17.5%	N/A	1:2–1:128 (50%–0.78%)
14	10⁵	5	2.90%	8%	52.3%	N/A	2.7%	N/A	N/A	1:2–1:128 (50%–0.78%)
15	10²	7	0.67%	*	*	*	*	*	*	1:2–1:64 (50%–1.56%)
16	10³	5	2.61%	*	*	*	*	*	*	1:2–1:64 (50%–1.56%)
17	10³	5	N/A	7%	50%	N/A	N/A	23%	N/A	1:2–1:16 (50%–0.6.25%)
18	10³	5	N/A	7.27%	N/A	36.37%	N/A	18.17%	N/A	1:2–1:16 (50%–0.6.25%)
19	10⁵	4	3.07%	5.62%	N/A	N/A	21.23%	21.05%	N/A	1:2–1:16 (50%–0.6.25%)
20	10³	8	1.29%	*	*	*	*	*	*	1:2–1:4 (50%–25%)

Note

N/A denotes a chemical not contained in a particular embalming solution.

* A chemical whose presence in a particular embalming solution is either not known or not shown in this table.

**FIGURE 1:**
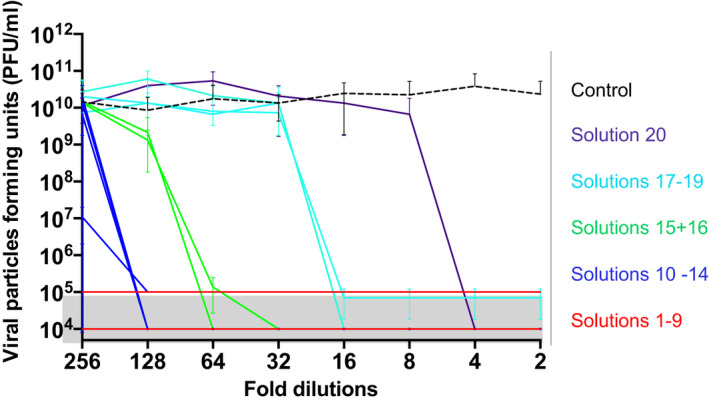
Schematic representation of the summary of the antiviral performance of the 20 embalming solutions tested at different dilutions starting from a 1:2 up to a 1:256 dilution. Solutions 1–9 (red) neutralised the SARS‐CoV‐2 at all dilutions tested, solutions 10–14 (blue) neutralised the SARS‐CoV‐2 up to a dilution of 1:128, solutions 15 and 16 (green) neutralised the SARS‐CoV‐2 up to a dilution of 1:64, solutions 17–19 (cyan) neutralised the SARS‐CoV‐2 up to a dilution of 1:16 and solution 20 (indigo) neutralised the SARS‐CoV‐2 up to a dilution of 1:4. For details on the content of each solution, see Table 1 and Table S1. For the detailed graphic representation of the performance of each embalming solution, see Figures S1 and S2

Almost half of the embalming solutions tested (nine out of 20, 45%) showed the ability to neutralise the virus (>3‐log^10^ reduction) at all dilutions (1:2–1:256, corresponding to solution concentrations of 50%–0.39% of the original solution that is used to embalm a body). Moreover, five out of 20 (25%) embalming solutions showed the ability to neutralise the virus at most dilutions (1:2–1:128, corresponding to 50%–0.78% of the original). Two solutions showed the ability to neutralise the virus at dilutions 1:2–1:64 (50%–1.56% concentration of the original), while four solutions showed less ability to neutralise the virus (three solutions 1:2–1:16, concentration 50%–6.25%, and one 1:2–1:4, concentration 50%–25%; Figure 1; Figures S1 and S2; Table 1). Interestingly, while embalming solutions with the low ability to neutralise the virus tended to have lower concentrations of formaldehyde, there were exceptions. These included for example solution 19 with a relatively high formaldehyde content and relatively lower efficacy against SARS‐CoV‐2, and solutions 4, 17 and 18 which do not contain any formaldehyde but showed efficient inhibitory activity (particularly solution 4 that performed also at very high dilutions). This may primarily be due to alternative ingredients such as glutaraldehyde (for solution 4) or phenol (for solutions 17 and 18).

These findings clearly demonstrate that all 20 solutions tested to neutralise the SARS‐CoV‐2 virus in vitro, 14 of them at very high dilutions (up to 256 or 128 times, i.e. 0.39% or 0.78% of the original solution). These data should inform decision‐making about the safety of embalming procedures for the resumption of body donor programmes. From a practical point of view, embalming involves perfusion of approximately 20–25 L of embalming fluid into an adult body, resulting in a dilution of approximately 1:4, that is a four‐fold dilution of the embalming solution. Therefore, in the context of optimal perfusion of the body with embalming solution, it is reasonable to suggest that the fluid that penetrates the body would remain at a higher concentration than the concentrations shown to inactivate the SARS‐CoV‐2 in the current study. Therefore, the embalming solutions tested in the current study are likely to neutralise SARS‐CoV‐2 in donor tissues when delivered using standard embalming procedures.

While this study provides significant reassurance that anatomical embalming solutions effectively neutralise SARS‐CoV‐2, it should be noted that donor bodies still need to be handled by technical personnel upon their arrival and during the embalming process. While this activity tends to involve small numbers of appropriately trained professionals, it is clear that suitable Personal Protective Equipment and safety measures, such as those normally employed for embalming to protect against agents such as HIV, hepatitis B and Ebola still need to be rigorously employed. Similarly, despite the high efficacy of the embalming solutions tested in neutralising SARS‐Cov‐2, we continue to urge caution in accepting bodies and avoid cases of suspected or certified SARS‐CoV‐2 infection. The current findings leave the question open of how to operate fresh‐frozen body donor programmes safely (such as those commonly used for surgical research and training; Hayashi et al., 2016), where donor material is not embalmed, given that SARS‐CoV‐2 remains stable and viable for longer at low temperatures (Aboubakr et al., 2021; Chin et al., 2020).

Interestingly, the soft embalming solutions tested, that is 4, 11, 15, 16, 17, 18, 20, also showed efficacy in neutralising the virus and these may represent a suitable alternative during the COVID era for anatomical units otherwise routinely using fresh frozen bodies. In particular, as noted above, some of them do not contain formaldehyde (4, 17 and 18) and their embalming potential relies on the inclusion of biocides (significant amounts of glutaraldehyde and phenol) that enable embalming and the neutralisation of SARS‐CoV‐2, without the tissue fixation associated with classical formalin‐based solutions.

In summary, our finding that all 20 anatomical embalming solutions tested effectively neutralise the SARS‐CoV‐2, and a number of them at concentrations much lower than they are normally used, provides a robust scientific basis upon which to make informed decisions about the safe resumption of body donor programmes and the subsequent deployment of embalmed donors for undergraduate and postgraduate teaching.

## CONFLICT OF INTEREST

The authors have no conflicts of interest.

## Supporting information

Fig S1

Fig S2

Table S1

## Data Availability

All data will be made freely available, upon request, from the authors.
