# Supplementary material for: Neutralisation of SARS‐CoV‐2 by anatomical embalming solutions
Source: J Anat. 2021 Oct 11;239(5):1221–5. doi: 10.1111/joa.13549 (PMC8546517; doi:10.1111/joa.13549)
Supplement: Supplementary file 1 — Fig S1 [file JOA-239-1221-s003.docx]

**Figure S1**. Graphic representation of the antiviral performance of the individual embalming solutions 1–10 tested at different dilutions starting from a 1:2 to a 1:256 dilution. Solutions 1–9 neutralised the SARS-CoV-2 at all dilutions tested, whereas solution 10 neutralised the SARS-CoV-2 in a range of dilutions from 1:2 to 1:128. For details on the content of each of these solutions, see Table 1.
