# Supplementary material for: Neutralisation of SARS‐CoV‐2 by anatomical embalming solutions
Source: J Anat. 2021 Oct 11;239(5):1221–5. doi: 10.1111/joa.13549 (PMC8546517; doi:10.1111/joa.13549)
Supplement: Supplementary file 2 — Fig S2 [file JOA-239-1221-s001.docx]

**Figure S2.** Graphic representation of the antiviral performance of the individual embalming solutions 11–20 tested at different dilutions starting from a 1:2 to a 1:256 dilution. Solutions 11–14 neutralised the SARS-CoV-2 in a range of dilutions from 1:2 to 1:128, solutions 15 and 16 neutralised the SARS-CoV-2 in a range of dilutions from 1:2 to 1:64, solutions 17–19 neutralised the SARS-CoV-2 in a range of dilutions from 1:2 to 1:16 and solution 20 neutralised the SARS-CoV-2 in a range of dilution 1:2–1:4. For details on the content of each of these solutions, see Table 1 and Supplementary Table 1.
