# Supplementary material for: Neutralisation of SARS‐CoV‐2 by anatomical embalming solutions
Source: J Anat. 2021 Oct 11;239(5):1221–5. doi: 10.1111/joa.13549 (PMC8546517; doi:10.1111/joa.13549)
Supplement: Supplementary file 3 — Table S1 [file JOA-239-1221-s002.docx]

| **Thiel Arterial Solution (95l batches)** | | | | |
| --- | --- | --- | --- | --- |
| **Chemical** | **g/l** | **ml/l** | **per 95L** | **%** |
| Boric acid | 17.9 |  | 1,700.5 | 1.79% |
| Ammonium nitrate | 117.9 |  | 11,200.5 | 11.79% |
| Potassium nitrate | 29.5 |  | 2,802.5 | 2.95% |
| Sodium sulphite | 49.5 |  | 4,702.5 | 4.95% |
| Propylene glycol |  | 209.5 | 19,902.5 | 20.95% |
| Chlorocresol | 3.5 |  | 332.5 | 0.35% |
| Formaldehyde |  | 12.9 | 1,225.5 | 1.29% |
| Ethanol |  | 69.5 | 6,602.5 | 6.95% |
| Morpholine |  | 10.5 | 997.5 | 1.05% |
| Water |  |  |  | 47.93% |
|  |  |  |  | 100.00% |

| **Thiel Venous Solution (49l batches)** | | | | |
| --- | --- | --- | --- | --- |
| **Chemical** | **g/l** | **ml/l** | **per 49L** | **%** |
| Boric acid | 14.9 |  | 730.1 | 1.49% |
| Ammonium nitrate | 96.3 |  | 4,718.7 | 9.63% |
| Potassium nitrate | 24 |  | 1,176.0 | 2.40% |
| Sodium sulphite | 35.2 |  | 1,724.8 | 3.52% |
| Propylene glycol |  | 179.6 | 8,800.4 | 17.96% |
| Chlorocresol | 3.5 |  | 171.5 | 0.35% |
| Formaldehyde |  | 26.1 | 1,278.9 | 2.61% |
| Ethanol |  | 203.6 | 9,976.4 | 20.36% |
| Morpholine |  | 20.4 | 999.6 | 2.04% |
| Water |  |  |  | 39.64% |
|  |  |  |  | 100.00% |

| **Tank Solution (2200L)** | | | | |
| --- | --- | --- | --- | --- |
| **Chemical** | **g/l** | **ml/l** | **per 2200L** | **%** |
| Boric acid | 27.3 |  | 60,060 | 2.73% |
| Ammonium nitrate | 90.9 |  | 199,980 | 9.09% |
| Potassium nitrate | 45.5 |  | 100,100 | 4.55% |
| Sodium sulphite | 61.4 |  | 135,080 | 6.14% |
| Propylene glycol |  | 108 | 237,600 | 10.80% |
| Chlorocresol | 1.7 |  | 3,740 | 0.17% |
| Formaldehyde | 6.7 |  | 14,740 | 0.67% |
| Ethanol |  |  |  |  |
| Morpholine |  |  |  |  |
| Water |  |  |  | 65.85% |
|  |  |  |  | 100.00% |
